# A Low-Cost AI-Empowered Stethoscope and a Lightweight Model for Detecting Cardiac and Respiratory Diseases from Lung and Heart Auscultation Sounds

**DOI:** 10.3390/s23052591

**Published:** 2023-02-26

**Authors:** Miao Zhang, Min Li, Liang Guo, Jianya Liu

**Affiliations:** 1 School of Mathematics, Shandong University, Jinan 250100, China; 2 School of Mathematics and Statistics, Shandong University, Weihai 264200, China; 3 Data Science Institute, Shandong University, Jinan 250100, China

**Keywords:** digital stethoscope, cardiac diseases, lung respiratory diseases, deep learning, random forest

## Abstract

Cardiac and respiratory diseases are the primary causes of health problems. If we can automate anomalous heart and lung sound diagnosis, we can improve the early detection of disease and enable the screening of a wider population than possible with manual screening. We propose a lightweight yet powerful model for simultaneous lung and heart sound diagnosis, which is deployable in an embedded low-cost device and is valuable in remote areas or developing countries where Internet access may not be available. We trained and tested the proposed model with the ICBHI and the Yaseen datasets. The experimental results showed that our 11-class prediction model could achieve 99.94% accuracy, 99.84% precision, 99.89% specificity, 99.66% sensitivity, and 99.72% F1 score. We designed a digital stethoscope (around USD 5) and connected it to a low-cost, single-board-computer Raspberry Pi Zero 2W (around USD 20), on which our pretrained model can be smoothly run. This AI-empowered digital stethoscope is beneficial for anyone in the medical field, as it can automatically provide diagnostic results and produce digital audio records for further analysis.

## 1. Introduction

According to a report of the WHO, cardiac and respiratory diseases are the primary causes of health problems, leading to the death of millions of people annually worldwide [1]. Early detection is the key factor in enhancing the effectiveness of intervention [1]. The stethoscope is a low-cost, yet efficient, auscultation device, which allows the assessment of cardiac and respiratory status through the evaluation of respiratory rate and effort, respiratory sounds, heart sounds, and heart rhythm [2]. However, auscultation heavily relies on a physician’s experience, which is a highly subjective process [3]. Sometimes, sound signals are highly complicated when detecting various heart or lung diseases [4]. Previous studies have reported the ambiguous identification and interpretation of sounds in auscultation as a generic issue in the clinical setting [5], which should not be neglected, as it may lead to inaccurate diagnosis and mistreatment [6]. Indeed, the European Respiratory Society, International Lung Sounds Association, and American Thoracic Society call for the standardization of the nomenclature of auscultation sounds [7].

Recently, there have been significant recent advances in applying deep learning analytics in interpreting human body sounds for clinical purposes [8]. If we can develop automated methods to detect such anomalous sounds, it will improve the early detection of disease and enable the screening of a wider population than possible with manual screening [9]. However, most previous studies have focused on separately training an independent model for lung or heart sound diagnosis. It is important to have a model that can simultaneously detect abnormal lung and heart sounds given that cardiac and respiratory diseases have many common features, such as cough, tachypnoea, dyspnoea, syncope, and cyanosis, which can make diagnosis problematic [3]; additionally, it is a common clinical procedure for physicians and nurses to conduct a holistic assessment of the respiratory and cardiac system. In addition, there is a need to develop a lightweight yet powerful model for lung and heart sound diagnosis that is deployable in an embedded low-cost device and is valuable in remote areas or developing countries where Internet access may not be available. Moreover, as COVID-19 sweeps the globe, an AI-empowered electronic stethoscope can lower the infection risk in medical workers. Therefore, it is imperative to leverage artificial intelligence to assist physicians and nurses in remotely and accurately conducting auscultation.

Our study aimed to fill in the gaps by contributing the following: Firstly, we developed a hybrid model that harnesses the power of CNN and best discrepancy forest (BDF, a variant of random forest) to classify cardiac and respiratory dysfunction. The experiments showed that the hybrid model outperforms the state-of-the-art methods. In addition, we designed a cost-effective digital stethoscope, which we connected to a low-cost Raspberry Pi Zero 2w single-board computer. The experiments showed that our proposed pretrained model ran smoothly on the computer. The cost of this AI-empowered stethoscope is so low (around USD 25) that it can be widely used in developing countries.

This article is organized as follows: Section 2 summarizes the related studies that have applied deep learning or machine learning techniques to classify lung or heart sounds. Section 3 describes our proposed model and the datasets. Section 4 shows the results and a evaluation of our model. Finally, Section 5 presents the conclusions and future work.

## 2. Related Work

### 2.1. Heart Sound Diagnosis

Artificial intelligence techniques have long been used to identify and classify heart diseases. Early works focused on traditional machine learning methods, for example, the naïve-Bayes-based electrocardiogram grating method proposed by Cheema and Singh [10], the SVM-based ventricular septal defects diagnosis method proposed by Sun et al. [11], and the rule-based classification tree proposed by Karar et al. [12]. Most of these machine learning methods achieved satisfactory accuracy in abnormal heart sound detection (on average, around 94%). Later on, people proposed neural network methods, for example, [13,14]. The average accuracy was around 90%. Recently, CNN-based methods have been built. For example, Deperlioglu [15] proposed an eight-layer CNN and achieved an accuracy of 97.90%. The ensemble CNN developed by Noman et al. achieved an accuracy of 89.22%. Yaseen et al. [16] used the mel frequency cepstral coefficient (MFCC) and discrete wavelet transform (DWT) to extract the features from heart sound signals and proposed a hybrid SVM and DNN model. Their model achieved an accuracy of more than 97%. Finally, Alqudah et al. [17] developed a new methodology using bispectrum higher-order spectral analysis and the CNN classification algorithm, which achieved an accuracy of 98.70%.

### 2.2. Lung Sound Diagnosis

Some of the pioneers of automatic lung sound diagnosis were Rocha et al. [18]. They extracted sound features (i.e. wheezes, crackles, or both) and then used machine learning models to perform classifications. However, there are two challenges in the field. The first one is that lung sound data are rare and their distribution is usually skewed across different classes. The second challenge is extracting useful features from soft breath sounds. For the first challenge, recent studies (for example, Mikolajczyk et al. [19], Nguyen et al. [20], and Lella [21]) have used data augmentation techniques, which not only add more training data to the model while resolving class imbalance issues but also improve model prediction accuracy and generalization ability. For example, Bardou et al. [22] achieved the highest satisfactory classification accuracy of approximately 97% with a large CNN model. For the second challenge, previous studies have also developed different feature extraction techniques. For example, Demir et al. [23] converted lung sounds to spectrogram images using the short-time Fourier transform method. Hai et al. used an optimized S-transform method. Shuvo et al. [24] used empirical mode decomposition and continue wavelet transform. Finally, previous studies have employed various classifiers ranging from machine learning methods (e.g., kNN, SVM, decision tree, and LDA, see [25]) to deep learning methods (e.g., CNN, CRNN, and ResNet) (see [26,27]).

## 3. Materials and Methods

Although previous deep learning models have achieved satisfactory performance, there is room for improvement. Firstly, none of these models can simultaneously treat heart and lung sound data. Secondly, these models are too large to be deployed to embedded devices. Finally, the classification performance of these models can be further increased with a new classifier. Motivated by the above-mentioned factors, we developed a lightweight hybrid model that leverages the power of CNN and ensemble learning. The experiments showed that the hybrid model is capable not only of diagnosing 11 types of heart and lung diseases with satisfactory performance but also of being deployed on a low-cost single-board computer.

The proposed methodology constitutes multiple steps:

Step 1: Both heart and lung sound data were acquired from two publicly available databases.

Step 2: The data were preprocessed with three methods (i.e., bandpass filtering, truncation, and normalization).

Step 3: The data were augmented to achieve balanced classes.

Step 4: The data were transformed into 2D bispectrum images.

Step 5: A lightweight hybrid model was developed, which constitutes a CNN model and a forest-based classifier.

Step 6: The image dataset was randomly split into two subsets: 80% as the training data and 20% as the test data.

Step 7: The hybrid model was trained with the training data.

Step 8: The hybrid model was tested with the test data, and multiple classification performance indicators were calculated.

Step 9: The hybrid model was deployed on a Raspberry PI Zero 2W single-board computer, which was connected to a digital stethoscope.

### 3.1. Dataset

We employed two publicly available datasets that are widely used as benchmark datasets for lung or heart sound diagnosis. The lung sound dataset used is the International Conference on Biomedical Health Informatics (ICBHI) 2017 dataset [28]. The dataset was independently collected from 126 subjects in Greece and Portugal. It contains 5.5 h of audio recordings sampled at different frequencies (4 kHz, 10 kHz, and 44.1 kHz). The length of the recordings ranges from 10 s to 90 s. The respiratory sounds are professionally annotated, while taking the following conditions into account: the subject’s pathological condition and the presence of respiratory anomalies (i.e., crackles and wheezes) in each respiratory cycle. The ICBHI samples include five classes: healthy (H), pneumonia (P), chronic obstructive pulmonary disease (COPD), bronchiolitis (BO), bronchiectasis (BA)m and upper respiratory tract infection (URTI).

In addition, the heart sound dataset we used is the one provided by Yaseen et al. [16]. It contains 1000 sound records that are evenly distributed in five main categories (i.e., 200 records per category): normal (N), aortic stenosis (AS), mitral stenosis (MS), mitral regurgitation (MR), and mitral valve prolapse (MVP). The heart sound records were collected from different sources and resampled to an 8000 Hz frequency rate and finally converted to a mono channel.

### 3.2. Data Preprocessing

We followed the best practices in previous studies to preprocess the two sound datasets. As mentioned in previous studies, auscultation signals generally reside in the frequency range of 25–400 Hz [9]. Every data file (i.e., signal sequence) in both the lung and heart sound dataset was first processed with a 2nd-order Butterworth bandpass filter with upper and lower cut-off frequencies of 25 and 400 Hz, respectively. Then, all the sample audio signals were resampled at 1000 Hz to ensure consistency while lowering the computational cost [8]. Next, every sound signal sequence was truncated to 2.5 s (i.e., the first 2500 data points, see [17,29,30]). Every signal sequence was normalized to (−1,1) in order to reduce the effect of device/sensor variation [27].

**Figure 1:**
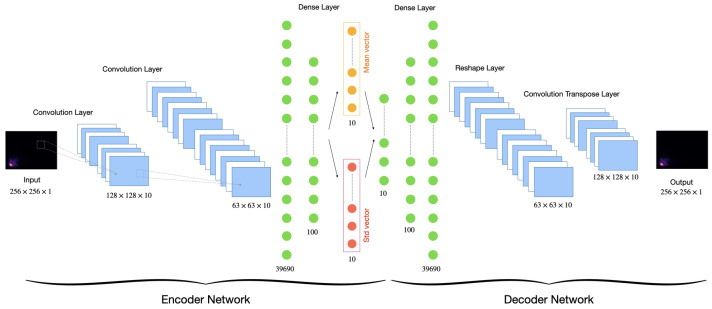
VAE scheme configuration for data augmentation.

Finally, we followed a previous approach [31] to employ a variation autoencoder (VAE) to solve the problem of imbalanced classes in the original datasets. The VAE used the mean and standard deviation layers to sample the latent vector (see Figure 1). The distribution of classes before and after data augmentation are represented in Table 1. Finally, the augmented dataset was used for our experiments.

### 3.3. Data Augmentation

The lung sound dataset is imbalanced, as one class label (i.e., COPD) has a very high number of observations and the other classes have very low numbers of observations. In addition, the heart sound dataset is relatively small (i.e., 200 samples per class). If both datasets are merged into a single one, then the distribution of the classes would be highly skewed.

The performance of a deep learning model particularly depends on the quality, quantity, and relevance of the training data. Given that collecting new lung and heart sound data is an exhausting and costly process, we leveraged data augmentation to make our proposed model more robust. We followed a previous approach [31] to employ a variation autoencoder (VAE) to solve the problem of imbalanced classes in the original datasets. The VAE uses the mean and standard deviation layers used to sample the latent vector (see Figure 1, for more details, see [31]). After data augmentation, the total number of samples was increased from 1917 to 8067. The distribution of classes before and after data augmentation is represented in Table 1. The 2 datasets were then combined into 1 with 11 evenly distributed classes.

### 3.4. Image Generation

Given that sound signals are nonstationary and non-Gaussian in nature, the bispectrum is one of the most widely used higher-order spectral analysis methods to generate images from sound [30]. The bispectrum quantifies the degree of quadratic phase coupling and nonlinearity interactions in nonstationary signals. A previous study [17] showed that the accuracy of the models based on full 2D bispectrum images is significantly higher than that of those based on contours.

**Figure 2:**
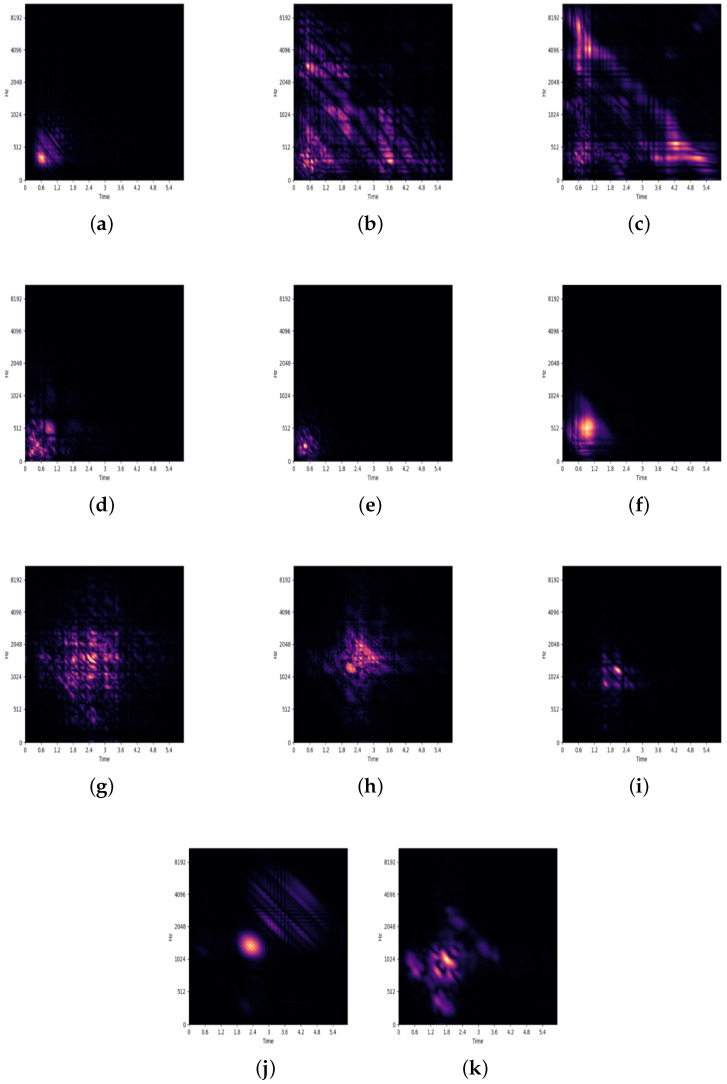
Generated bispectrum illustrations for every category of the lung and heart sound records. (**a**) BA, (**b**) BO, (**c**) COPD, (**d**) H, (**e**) P, (**f**) URTI, (**g**) AS, (**h**) MR, (**i**) MS, (**j**) MVP, and (**k**) N.

**Figure 3:**
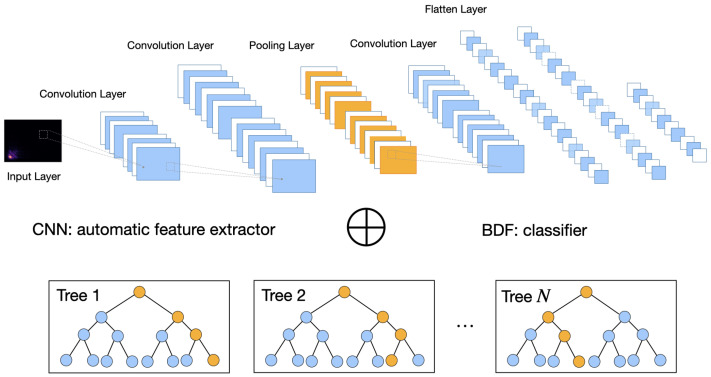
Architecture of the hybrid model.

Therefore, we followed prior studies [29,32] to define the bispectrum of a sound signal with the second-order Fourier transform with the third-order cumulants of the signal. That is, the bispectrum expresses the nature of a sound record as an image to extract the most represented features for each class [33,34]. We computed the full 2D bispectrum images of all sound records after data augmentation. The resultant images, each of which was 256×256 pixels, were stored in an image database with their class labels. We demonstrate a few samples of the 11 classes in Figure 2. The image database is publicly available on Github https://github.com/DataScienceSDU/Heart-Lung-Sound (accessed on 21 January 2023). Figure 3 illustrates how a sound record is transformed into an image. The image database is publicly available.

### 3.5. Model Proposition

Building on the work of Tariq et al. [8,35], we developed a lightweight hybrid model by adjusting the network structure and parameters and by replacing the last fully connected layer with a best discrepancy forest classifier. Figure 3 illustrates the architecture of the hybrid model.

The hybrid model consists of two parts. The first part is a 2D CNN structure for feature extraction. The 2D CNN is composed of six layers, as shown in Table 2:

- The input layer is set to 256×256.
- The first 2D convolutional layer takes the bispectrum as the input with 24 filters. The kernel size is set to 5×5 with a stride of 4×2 and with ReLU as the activation function.
- The second 2D convolutional layer has 48 filters. The kernel size is set to 5×5 with a stride of 1×1.
- Thirdly, a 2D max-pooling layer is set up with a 4×2 kernel and a 4×2 stride.
- Finally, a 2D convolutional is set up with 16 filters. The kernel size is set to 3×3 with a stride of 1×1.

As shown in Table 2, there were only 36,400 parameters in our proposed CNN that needed to be estimated. This is much smaller than that proposed in previous studies [8,35]. We intended to keep the CNN relatively small so that our proposed model can be deployed in embedded devices that are typically computational-resource-constrained.

After the high-level features are extracted through the convolutional and pooling operations, the output feature maps are transformed into a 1D vector and transferred to a fully connected layer with 64 neurons.

The fully connected layer is connected to the second part of the hybrid model, a best discrepancy forest (BDF) classifier, which is a variant of random forest (RF), with 500 trees [36]. Like RF, BDF combines bagging and random selection of features in order to construct a collection of decision trees (i.e., 500 trees in this study) with controlled variance.

Bagging means “bootstrap aggregating”. Given a training dataset *D* with *N* observations, bagging generates *m* new training sets $D_{i}$, each of size *n*, where n<N, by sampling from *D* randomly (RF) or systematically (BDF) and with replacement [36]. Each new training set $D_{i}$ is used to train a single decision tree. The only difference between the BDF and the RF is the way in which *n* observations are selected.

The RF uses the simple random sampling technique with replacement while the BDF uses the systematic sampling technique with replacement [37]. The systematic sampling is proven mathematically and empirically by the work [37] that it can make sure that the distribution of selected *n* observations within each new training sets $D_{i}$ is similar to that of the whole dataset *D* (please refer to [37] for the mathematical proof and empirical experimental results with 160 datasets).

Our proposed model uses a BDF of 500 trees. That is, the 64 neurons of the first part CNN serve as the input of the BDF classifier. By constructing 500 trees to form a “forest”, the predictions of all trees are aggregated to identify the most popular result of classification. The proposed model is illustrated in Figure 3.

### 3.6. Model Training and Testing

We followed the hold-out method by setting a random seed and then randomly split the image data generated in Section 3.4 into a training dataset (80% or 6453 images) and a test dataset (20% or 1614 images). The proposed model was first trained and then was tested with the corresponding datasets on a workstation with NVIDIA Telsa T4 GPU card of 16 GB display memory. All the systems were implemented using Tensorflow 2, using the Adam optimizer with 100 epochs, a mini batch size of 128, and cross-entropy loss. Then, the trained model was tested with the test dataset. The results in Table 3 show that, on average, our 11-class prediction hybrid model achieved 99.97% accuracy, 99.89% F1 score, 99.90% precision, 99.99% specificity, and 99.88% sensitivity. The confusion matrix shown in Table 4 indicates that among the 1614 testing samples, the hybrid model wrongly classified only two samples.

To make sure that the results were not achieved by accident, we conducted a robustness check and re-evaluated the proposed model with 10-fold cross-validation. That is, the image data were randomly split into ten partitions. We used nine of those partitions for training and reserve the tenth for testing. We repeated this procedure ten times, each time reserving a different tenth for testing. The means of every performance indicator are summarized in Table 3. We concluded that the results of the 10-fold cross-validation were highly similar to those of the hold-out validations (i.e., 99.94% accuracy, 99.72% F1 score, 99.84% precision, 99.89% specificity, and 99.66% sensitivity).

Finally, in order to verify whether the BDF classifier is really effective, we retrained and retested a pure CNN model without the BDF classifier. That is, the last layer of 64 neuros was directly connected to 11 classes. The pure CNN model was first trained and tested with the same hold-out datasets (i.e., 80% or 6453 images for training and 20% or 1614 images for test). The results in Table 3 show that, on average, the 11-class pure CNN model achieved 99.81% accuracy, 99.01% F1 score, 99.13% precision, 99.89% specificity, and 98.92% sensitivity. The confusion matrix shown in Table 4 indicates that, among the 1614 testing samples, the pure CNN model wrongly classified 17 samples. The pure CNN was also trained and tested with 10-fold cross validation. The results summarized in Table 3 indicate that it achieved 99.53% accuracy, 97.46% F1 score, 97.68% precision, 99.74% specificity, and 97.33% sensitivity. We also concluded that the hybrid model (i.e., CNN+BDF) outperformed the pure CNN model, especially in terms of F1 score, precision, and sensitivity.

## 4. Model Deployment with Edge Computing

We aimed to develop a lightweight yet powerful model that can assist practitioners in conducting lung and heart sound diagnoses with ordinal or digital stethoscopes. If the proposed deep learning models could be directly deployed on edge devices, then it would be possible to perform automated diagnosis and health care services at a distance.

**Figure 4:**
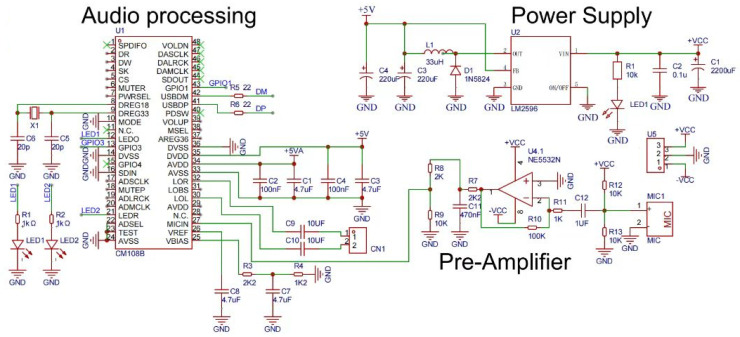
Schematic diagram of the proposed digital stethoscope.

We transformed an ordinary stethoscope into a digital one as follows: We first cut the tube that connects to the disc-shaped resonator in half to fit an electret condenser microphone (CMC-9745-44P). The microphone captured the signals of lung or heart sounds. The signals were then amplified through an amplifier (NE5532N) with an op-amp. Then, the signals were converted into digital ones through an analog-to-digital converter (CM108B). Finally, the converter was connected to a Raspberry PI Zero 2W (but could be connected to any other single-board/low cost computer) through a micro-USB port. An SPI LED screen was connected to the Raspberry Pi for displaying the diagnosis result. The schematic diagram of the digital stethoscope is shown in Figure 4 and the bill of materials (BOM) is listed in Table 5.

We now demonstrate how to deploy the pretrained proposed hybrid model into a Raspberry PI computer to make inferences. Firstly, given that the proposed hybrid model has two parts (i.e., CNN part to extract high-level features and BDF classifier), we converted the pretrained CNN model into a TFLite model through Tensorflow’s TFLiteConverter module and saved the pretrained BDF model through the Joblib module.

Then, we installed the relevant packages (i.e., TFLite Runtime, scikit-learn, soundfile, and libsndfile) on the Raspberry PI computer. We used the arecord command to record a 15 s sound through the USB-connected digital stethoscope and saved the sound signals a .wav file. We used the soundfile library to convert the .wav file into a Numpy array and then conducted the preprocessing analysis specified in Section 3.2. The resulting normalized signal was then converted to a 2D bispectrum image (i.e., a 256×256 matrix) using the method specified in Section 3.4. The image was fed into the pretrained CNN TFLite model to extract high-level features and then into the pretrained BDF for classification. The classification result was shown on the SPI LED screen. We conducted 10 experiments on a Raspberry PI Zero 2W. On average, the whole inference process required around nine seconds and consumes around 27.79% of 512M memory.

## 5. Discussion

This study contributes to the literature in the following ways: Firstly, we developed a new hybrid model that can simultaneously detect lung and heart diseases. Note that classification problems with many classes with imbalanced datasets present more of a challenge a problem with fewer classes. The experiments showed that our proposed hybrid model that deals with 11 classes can achieve better performance than other relevant models that deal with fewer classes using the two same datasets (see Table 3). For example, with the five-class heart sound dataset, Yaseen et al. [16] achieved 97.90% accuracy and 94.50% sensitivity; Glosh et al. [38] achieved 98.33% accuracy and sensitivity; and Alqudah et al. [29] achieved 98.70% accuracy and sensitivity.

With the six-class lung sound dataset (ICBHI), Fraiwan et al. [26] achieved 99.62% accuracy and 98.56% F1 score; Pham et al. [9] achieved 98.2% sensitivity and 84% F1 score; and Shuvo et al. [24] achieved 98.7% accuracy and 98.6% sensitivity.

Secondly, our findings confirm the those of prior studies [39,40,41] that ensemble learning classifiers (i.e., BDF in this study) can solve the over-fitting problem because, on the one hand, ensemble learning maximizes the diversity through the random selection of high-level input features extracted from the CNN part of our hybrid model; on the other hand, the bootstrap bagging mechanism can increase the strength among multiple decision trees and improve classification performance [36].

Finally, our proposed hybrid model is capable of being deployed in a low-cost single-board computer. Connecting the computer to a digital stethoscope through a mini-USB port can make automation in lung and heart disease diagnosis possible. Our work supports the claim that AI systems have the potential to improve diagnostic efficiency while reducing human errors in medicine [42]. Our findings also reveal that harnessing the power of edge computing can transform the healthcare field, as with most other industries, which offers unprecedented occasions to improve patient and clinical group results, decrease costs, and so on.

However, our study has a limitation. Although our proposed hybrid model has achieved satisfactory results in rigorous cross-validation experiments, we have not tested it in hospitals. This is because the cost of large-scale clinical data is relatively high, and it was difficult for us to work with patients under the high-risk conditions of COVID-19 infection. In the future, we hope to obtain additional research funding to produce numerous digital stethoscopes and to collaborate with hospital staff on new data acquisition and testing of our model.

## 6. Conclusions

Since its earliest appearance in 1816 as an impromptu paper cone rolled by Dr. René Laennec, designs for stethoscopes have the familiar consensus configuration: a chest piece, a pair of earpieces, and a tube or tubes connecting them. The stethoscope continues to play an important role in the digital age. In this study, we developed a hybrid model that harness the power of CNN and the random forest classifier. The experiments confirmed the superiority of the proposed model, which not only achieves satisfactory performance but is also lightweight enough to be deployed in a low-cost single-board computer to form a digital stethoscope. Therefore, our study and the AI-empowered stethoscope solution are particularly important for people in remote areas and developing countries or practitioners involved in humanitarian relief.

## Figures and Tables

**Table 1:** Number of samples per class before and after augmentation.

Type	Class	Original	After
Heart Sound	AS	200	800
	MS	200	800
	MVP	200	800
	MR	200	800
	N	200	800
Lung Sound	COPD	793	793
	P	37	667
	H	35	665
	URTI	23	653
	BA	16	646
	BO	13	643

**Table 2:** Structure and parameters of proposed CNN model.

No.	Layer	Information		Param
1	Input layer	Size	256 × 256	
2	Conv2D	Number of filters	24	624
		Kernel size	5 × 5	
		Stride	4 × 2	
		Activation	RELU	
3	Conv2D	Number of filters	48	28,848
		Kernel size	5 × 5	
		Stride	1 × 1	
4	MaxPooling2D	Kernel size	4 × 2	
		Stride	4 × 2	
5	Conv2D	Number of filters	16	6928
		Kernel size	3 × 3	
		Stride	1 × 1	
		Activation	RELU	

**Table 3:** Performance expressed in percent of the hybrid model and of the pure CNN model based on 8:2 hold-out and 10-fold cross validation with comparisons.

	Model	Accuracy	F1 Score	Precision	Specificity	Sensitivity
8:2 Hold-Out	CNN+BDF	99.97%	99.89%	99.90%	99.99%	99.88%
	CNN	99.81%	99.01%	99.13%	99.89%	98.92%
10-Fold CV	CNN+BDF	99.94%	99.72%	99.84%	99.89%	99.66%
	CNN	99.53%	97.46%	97.68%	99.74%	97.33%
Pr. Studies Heart	Yaseen et al. [16]	97.90%				94.50%
	Glosh et al. [38]	98.33%				98.33%
	Alqudah et al. [29]	98.70%				98.70%
Pr. Studies Lung	Fraiwan et al. [26]	97.62%	98.56%			
	Pham et al. [9]	98.2%	84.0%			
	Shuvo et al. [24]	98.7%				98.6%

**Table 4:** Confusion matrix of 11 classes based on 20% of test dataset.

	BA	BO	COPD	H	P	URTI	AS	MR	MS	MVP	N
BA	121/120	0/0	0/0	0/0	0/0	0/0	0/0	0/0	0/0	0/0	0/0
BO	0/0	125/125	0/0	0/0	0/0	0/0	0/0	0/0	0/0	0/0	0/0
COPD	0/1	0/0	179/179	1/5	1/4	0/2	0/0	0/0	0/0	0/0	0/0
H	0/0	0/0	0/0	144/140	0/1	0/1	0/0	0/0	0/0	0/0	0/0
P	0/0	0/0	0/0	0/0	148/144	0/0	0/0	0/0	0/0	0/0	0/0
URTI	0/0	0/0	0/0	0/0	0/0	133/130	0/0	0/0	0/0	0/0	0/0
AS	0/0	0/0	0/0	0/0	0/0	0/0	155/154	0/0	0/1	0/1	0/0
MR	0/0	0/0	0/0	0/0	0/0	0/0	0/0	158/158	0/0	0/0	0/0
MS	0/0	0/0	0/0	0/0	0/0	0/0	0/0	0/0	148/147	0/0	0/0
MVP	0/0	0/0	0/0	0/0	0/0	0/0	0/1	0/0	0/0	156/155	0/0
N	0/0	0/0	0/0	0/0	0/0	0/0	0/0	0/0	0/0	0/0	145/145

Note: CNN+BDF/CNN.

**Table 5:** Bill of materials of the electronic stethoscope.

No.	Quantity	Comment	Designator	Footprint
1	4	4.7 uF	C1, C3, C7, C8	C0603
2	1	2200 uF	C1	CP_16X25MM
3	2	100 nF	C2, C4	C0603
4	1	0.1 u	C2	C0805
5	2	220 uF	C3, C4	CAP-TH_BD8.0-P3.50-D1.0-FD
6	2	20 p	C5, C6	C0603
7	2	10 UF	C9, C10	C0603
8	1	470 nF	C11	C0603
9	1	1 UF	C12	C0603
10	1	XH2.54*2P	CN1	CONN-TH_2P-P2.50-XH2.54-2P
11	1	1N5824	D1	SMA/DO-214AC
12	1	33 uH	L1	IND-SMD_L7.7-W7.3
13	2	0805G (green)	LED1, LED2	led0805
14	1	LED-0805_R	LED1	LED0805_RED
15	1	MIC	MIC1	MIC-TH_BD6.0-P2.00
16	2	1 kΩ	R1, R2	R0603
17	1	10 k	R1	R0805
18	2	2K2	R3, R7	R0603
19	1	1K2	R4	R0603
20	2	22	R5, R6	R0603
21	1	2 K	R8	R0603
22	3	10 K	R9, R12, R13	R0603
23	1	100 K	R10	R0603
24	1	1 K	R11	R0603
25	1	CM108B	U1	LQFP-48_L7.0-W7.0-P0.50-LS9.0-BL
26	1	LM2596	U2	TO-263-5_L10.6-W9.6-P1.70-LS15.9-BR
27	1	NE5532N	U4	DIP-8_L9.3-W6.4-P2.54-LS7.6-BL
28	1	XH2.54-WI-3P	U5	CONN-TH_XH2.54-WI-3P
29	1	8 MHZ 20 PF 10 PPM	X1	OSC-SMD_L5.0-W3.2

## Data Availability

The image database is publicly available on Github (https://github.com/DataScienceSDU/Heart-Lung-Sound) (accessed on 21 January 2023).
